# Higher incubation temperatures produce long-lasting upward shifts in cold tolerance, but not heat tolerance, of hatchling geckos

**DOI:** 10.1242/bio.042564

**Published:** 2019-04-15

**Authors:** Theja Abayarathna, Brad R. Murray, Jonathan K. Webb

**Affiliations:** School of Life Sciences, University of Technology Sydney, Broadway, New South Wales 2007, Australia

**Keywords:** Heat wave, Developmental plasticity, Critical thermal limits, Thermal tolerance, Lizard

## Abstract

Heatwaves are a regular occurrence in Australia, and are predicted to increase in intensity and duration in the future. These changes may elevate temperatures inside lizard nests, shortening the incubation period, so that hatchlings are more likely to emerge during heatwaves. Potentially, developmental plasticity or heat hardening could buffer hatchings from future warming. For example, higher incubation temperatures could shift critical thermal maxima upwards, enabling lizards to withstand higher temperatures. To investigate whether developmental plasticity affects hatchling thermal tolerance, we incubated eggs of the velvet gecko *Amalosia lesueurii* under two fluctuating incubation treatments to mimic current (mean=24.3°C, range 18.4–31.1°C) and future ‘hot’ (mean=28.9°C, range 19.1–38.1°C) nest temperatures. We maintained the hatchlings under identical conditions, and measured their thermal tolerance (CT_max_) aged 14 days and 42 days. We then released hatchlings at field sites, and recaptured individually marked lizards aged 6 months, to determine whether incubation induced shifts in thermal tolerance were transitory or long-lasting. We found that at age 14 days, hatchlings from hot-temperature incubation had higher CT_max_ [mean=39.96±0.25°C (s.d.)] than hatchlings from current-temperature incubation [mean=39.70±0.36°C (s.d.)]. Hatchlings from the current-incubation treatment also had significantly higher heat hardening capacity [mean=0.79±0.37°C (s.d.)] than hatchlings from hot-temperature incubation treatment [mean=0.47±0.17°C (s.d. )]. However, both of these incubation-induced effects did not persist into later life. By contrast, incubation treatment had significant and long-lasting effects on the cold tolerance of hatchlings. At age 14 days, current-incubated hatchlings tolerated colder temperatures [CT_min_=11.24±0.41°C (s.d.)] better than hot-incubated hatchlings [CT_min_=14.11±0.25°C (s.d.)]. This significant difference in cold tolerance persisted into the juvenile life stage, and was present in 6-month-old lizards that we recaptured from field sites. This finding indicates that upward shifts in cold tolerance caused by higher incubation temperatures might affect overwinter survival of lizards, but field studies linking fitness to thermal tolerance are necessary to test this idea. Overall, our results suggest that developmental plasticity for heat tolerance is unlikely to buffer lizard populations from higher temperatures.

This article has an associated First Person interview with the first author of the paper.

## Abstract (precis)

**Summary:** Hatchling geckos from current temperature incubation tolerated cold better than hatchlings from future temperature incubation. The developmental shift in cold tolerance persisted for 6 months, and could therefore influence hatchling survival.

## INTRODUCTION

An understanding of how organisms cope with heatwaves can help to predict how future climatic changes may affect populations. Heatwaves are predicted to increase in intensity and duration in the future, and can have major effects on populations via direct mortality (Welbergen et al., 2008), or through more complex interactions with early life stages. Lizards are particularly sensitive to acute temperatures because their physiology, behaviour and locomotor performance is strongly dependent on body temperatures (Huey, 1982). Although juvenile and adult lizards can avoid extreme temperatures by selecting appropriate microhabitats (Huey, 1982), sessile life stages (eggs) are particularly vulnerable to exposure to extreme temperatures because embryos cannot thermoregulate (Telemeco et al., 2016). In most lizard species, females lay eggs in shallow underground nests where the developing embryos can experience thermal spikes during extreme heat events (Shine et al., 2003; Telemeco et al., 2009). While chronic exposure to high temperatures (typically >42°C) can result in embryo mortality (Angilletta et al., 2013; Levy et al., 2015), the effects of exposure to high, but not lethally high temperatures, on embryos and offspring have received less study.

Phenotypic plasticity may reduce the vulnerability of early life stages to extreme heat events. Maternal plasticity in nest site selection (choosing shadier nests) or the timing of oviposition (nesting earlier) could reduce the exposure of developing embryos to high temperatures (Urban et al., 2014). Even if females only partially compensate for increases in nest temperatures, developmental plasticity and acclimation may also affect the physiological traits of offspring in ways that increase fitness. For example, in several species of ectotherms, exposure of embryos to higher developmental temperatures may confer higher thermal tolerance in later life stages (Slotsbo et al., 2016; van Heerwaarden et al., 2016). Most of these studies have involved *Drosophila*, but concordant results have been demonstrated for other taxa (Sgro et al., 2016), suggesting that developmental plasticity for thermal tolerance may be widespread. Over shorter time periods, heat hardening, the process whereby individuals increase their heat tolerance after brief exposure to high temperatures, may provide fitness benefits to ectotherms during summer heatwaves (Hoffmann et al., 2003). For example, in *Drosophila melanogaster* heat hardened flies that were released during hot weather had significantly higher rates of recapture than control flies, suggesting that heat hardening conferred an advantage during hot conditions (Loeschcke and Hoffmann, 2007).

While developmental plasticity and heat hardening may help to buffer lizard populations from heatwaves, few studies have investigated how incubation temperatures influence the thermal tolerance or heat hardening capacity of hatchlings (Llewelyn et al., 2018; Noble et al., 2018; While et al., 2018). Moreover, it is not clear whether such effects, if present, persist into later life. For example, lizards may show ontogenetic shifts in thermal tolerance, and can exhibit longer-term acclimation to the changing environments in the field (Bowler, 2005). Such acclimation might swamp any effects of developmentally induced changes in thermal tolerance. To address these knowledge gaps, we investigated whether exposure to higher developmental temperatures likely to be experienced in the future affected the thermal tolerance of hatchling lizards. We also asked whether developmental temperatures influenced the heat hardening responses of hatchlings. To determine whether developmental effects were transient or long-lasting, we measured the thermal tolerance of hatchlings within 2 weeks (14 days) of birth, and after 6 weeks (42 days), before releasing them to field sites. To determine whether incubation-induced changes in thermal tolerance persisted into later life, we recaptured lizards from the field 4 months after release (at age 6 months), brought them back to the lab, and measured their thermal tolerance.

## RESULTS

### Effects of incubation temperature on hatching success, incubation period and body size

Hatching success was higher in the current-treatment (34 of 84 eggs hatched) than the hot-treatment (18 of 81 eggs hatched, Pearson chi-square=6.37, *P*=0.12). Incubation treatment also affected the incubation period; hot-incubated lizards were born, on average, 26 days earlier (mean incubation period=65.4 days, range 61–70 days) than current-incubated lizards (mean incubation period=91.6 days, range 73–101 days; two-factor ANOVA, incubation *F*_1,48_=181.6, *P*=0.0001; location *F*_1,48_=0.008, *P*=0.93, interaction *F*_1,48_=1.49, *P*=0.23). Hot-incubated lizards were also smaller in snout-vent length [mean=21.4±1.82 mm (s.d.)] than current-incubated lizards [mean=25.4±2.07 mm (s.d.); ANOVA, incubation *F*_1,48_=48.6, *P*=0.001; location *F*_1,48_=1.76, *P*=0.19; interaction *F*_1,48_=0.78, *P*=0.38], and were also lighter [mean=0.31±0.06 g (s.d.)] than current-incubated lizards [mean=0.40±0.06 g (s.d.), ANOVA, incubation *F*_1,48_=40.73, *P*=0.0001; location *F*_1,48_=0.15, *P*=0.70, interaction *F*_1,48_=0.25, *P*=0.62].

### Effects of incubation temperature on thermal tolerance

**Fig. 1.**
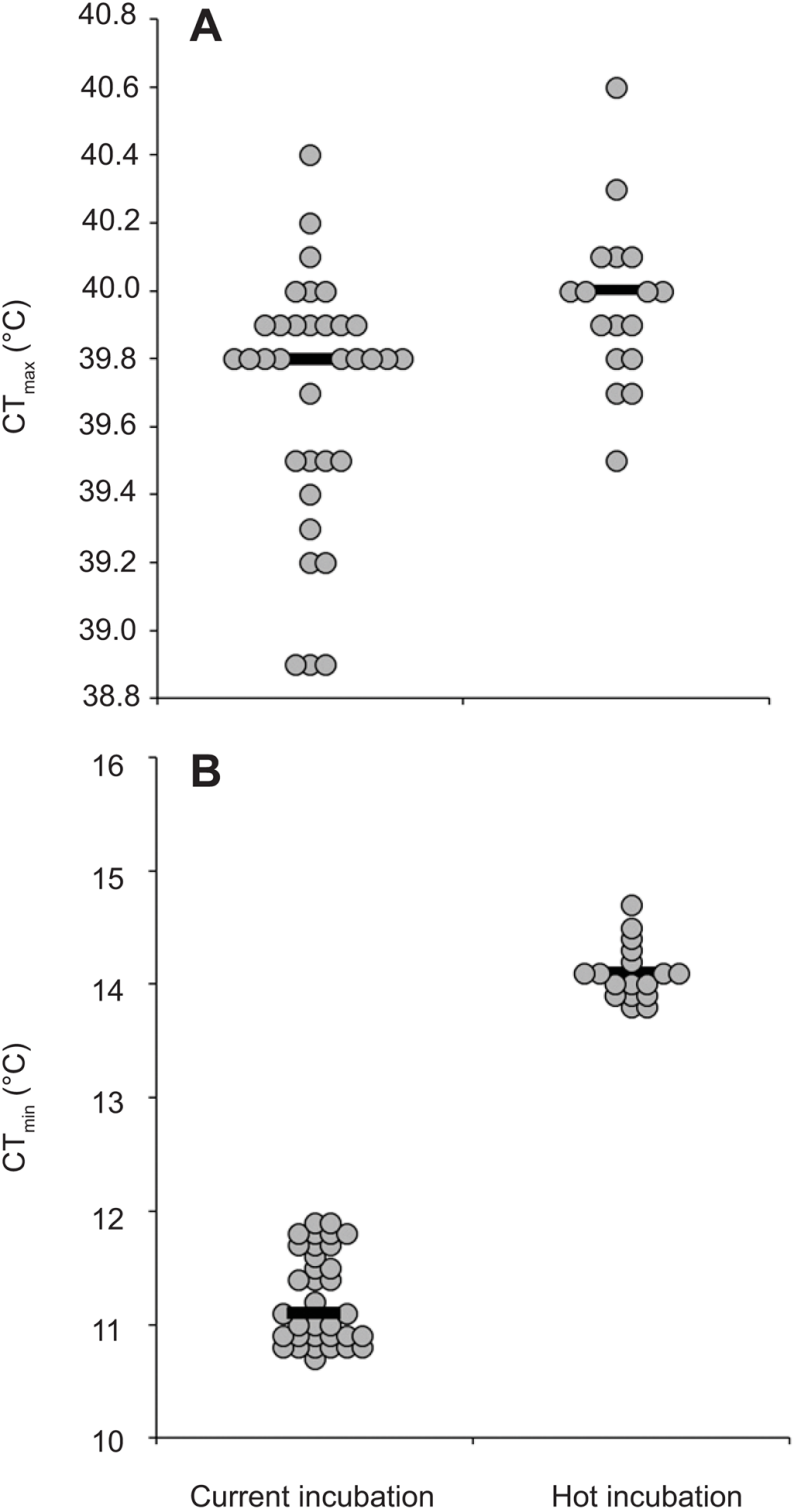
**Thermal tolerance of hatchling velvet geckos.** Dot plots of (A) maximum critical thermal tolerance (CT_max_) and (B) minimum critical thermal tolerance (CT_min_) of 14-day-old hatchling geckos from current (*n*=34) and hot (*n*=17) incubation treatments. Treatment means were significantly different in both panels (linear mixed effect models, *P*<0.01). Black lines show medians and repeat values are jittered for clarity.

Hot-incubated hatchlings had a higher CT_max_ than current-incubated hatchlings (39.96°C versus 39.70°C; t_23.27_=3.12, *P*=0.005; Fig. 1A). Random factors in the model were associated with very little (mother identity within location: variance±s.d.=0.04±0.20) to none (location) of the variation in CT_max_. Hot-incubated lizards also had a higher CT_min_ than current-incubated lizards (14.11°C versus 11.24°C; t_32.22_=27.59, *P*<0.0001, Fig. 1B). Random factors in the model explained very little (mother identity within location: variance±s.d.=0.02±0.15) to none (location) of the variation in CT_min_.

### Effects of incubation temperature on heat hardening

**Fig. 2.**
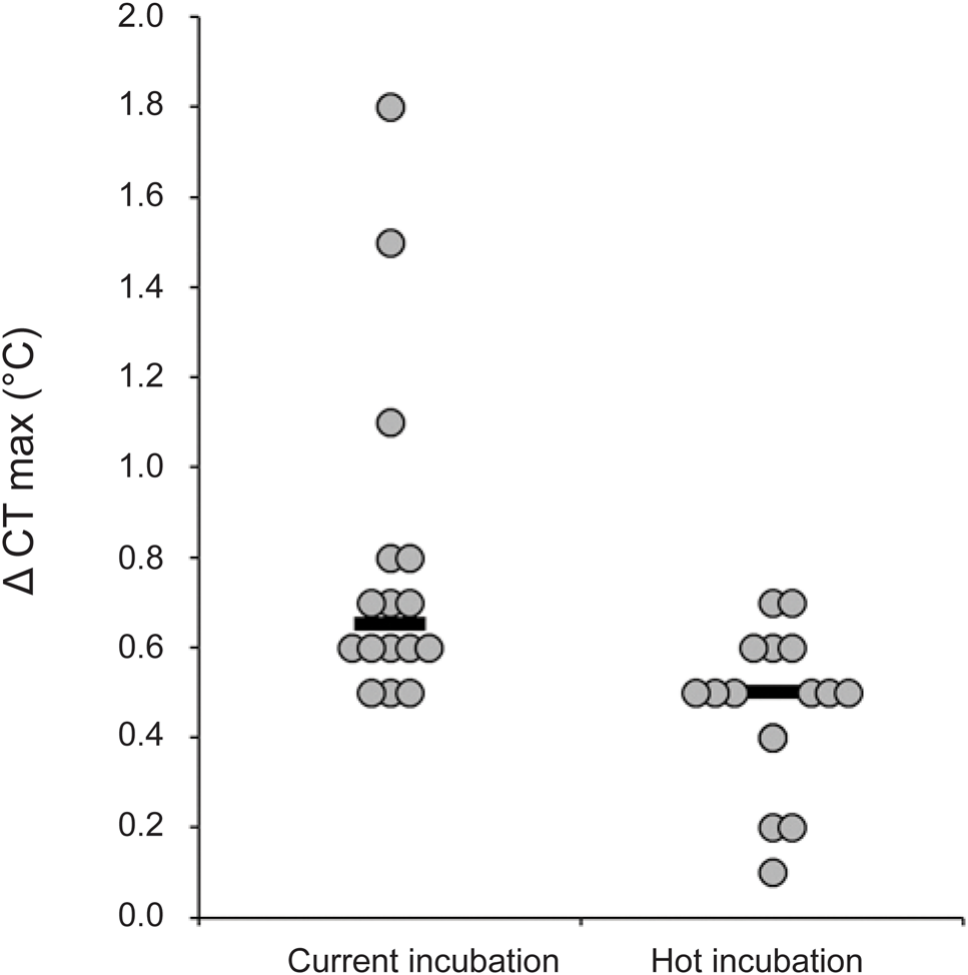
**Heat hardening dot plots for 6-week-old hatchling velvet geckos from current (*n*=16) and hot (*n*=16) incubation treatments.** Treatment means were significantly different (linear mixed effect model, *P*=0.002). Black lines show medians and repeat values are jittered for clarity.

Hot-incubated hatchlings also demonstrated reduced heat hardening compared with current-incubated hatchlings (0.47°C versus 0.79°C; t_29.58_=3.41, *P*=0.002; Fig. 2). Random factors in the model were associated with very little (location: variance±s.d.=0.01±0.09) to none (mother identity within location) of the variation in Δ CT_max_. We found no significant correlation between initial CTmax and Δ CT_max_ (Pearson correlation r=−0.54, *P*=0.77).

### Persistence of incubation induced shifts in thermal tolerance

**Fig. 3.**
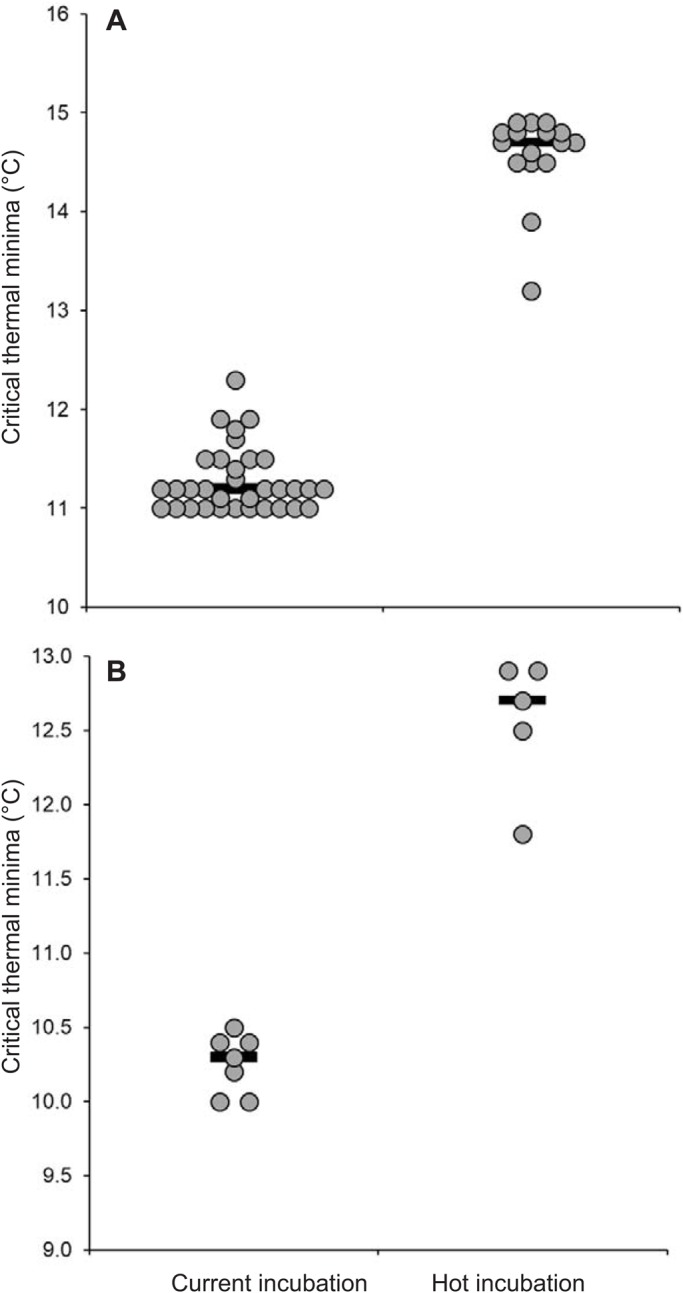
**Cold tolerance of velvet geckos from the two incubation treatments.** Dot plots of the critical thermal minima (CT_min_) of (A) 6-week-old (*n*=49) and (B) 6-month-old (*n*=12) hatchling geckos from current- and hot-incubation treatments. Treatment means were significantly different in A (linear mixed effect model, *P*<0.0001) and B (ANOVA, *P*<0.001). Note that 6-month-old geckos were recaptured in the field at the Dharawal study site. Black lines show medians and repeat values are jittered for clarity.

At age 6 weeks, there was no difference in the CT_max_ of hot-incubated or current-incubated lizards (40.02°C versus 39.88°C; t_46.92_=1.51, *P*=0.14), but current-incubated lizards had significantly lower CT_min_ (11.28°C) than hot-incubated lizards (14.58°C) (t_46.48_=30.22, *P*<0.0001, Fig. 3A). In mid-July, we systematically searched our field sites for individually marked lizards. We recaptured three current-incubated lizards from Nowra, and seven current-incubated and five hot-incubated lizards from Dharawal. Given the low sample size for Nowra, we could only analyse data for lizards from Dharawal. For these juveniles, we found no significant difference in CT_max_ of current-incubated [mean=39.9±0.21°C (s.d.)] or hot-incubated [mean=40.2±0.21°C (s.d.)] lizards at age 6 months (ANOVA: *F*_1,10_=3.5, *P*=0.09). Likewise, incubation treatment did not influence heat hardening capacity of current-incubated [mean=0.51±0.146°C (s.d.)] or hot-incubated lizards [mean=0.30±0.152°C (s.d.); ANOVA *F*_1,10_=0.09, *P*=0.07]. However, current-incubated lizards had lower CT_min_ [mean=10.24±0.22°C (s.d.)] than hot-incubated lizards [mean=12.56±0.46°C (s.d.), *F*_1,10_=144.47, *P*<0.0001, Fig. 3B].

## DISCUSSION

Developmental plasticity coupled with short-term heat hardening could potentially buffer lizards from the effects of summer heatwaves. In this study, we subjected developing embryos of the velvet gecko to thermal profiles that mimicked temperatures of currently used natural nests (current incubation treatment), and temperatures likely to occur during summer heatwaves in 2050 (hot incubation treatment). We found that hot-incubated hatchlings had significantly higher CT_max_ than current-incubated hatchlings, demonstrating that exposure to higher developmental temperatures shifted thermal tolerance upwards. This finding agrees with results from studies on *Drosophila* which found that flies reared at higher temperatures had higher heat tolerance than flies reared at lower temperatures (van Heerwaarden et al., 2016; Hoffmann et al., 2003; Slotsbo et al., 2016). However, in the *Drosophila* studies, developmental plasticity resulted in increases in heat tolerance of up to 1°C. By contrast, developmental shifts in heat tolerance in velvet geckos were small, and thus may confer little benefit to individuals.

Nonetheless, 6-week-old geckos exhibited clear heat hardening responses 4 h after exposure to high temperatures, with some individuals increasing their heat tolerance by up to 1.8°C (Fig. 2). Current-incubated geckos had significantly higher hardening capacity (mean=0.79±0.09°C) than hot-incubated geckos (mean=0.47±0.04°C). To date, few studies have measured heat hardening in lizards (Llewelyn et al., 2018; Phillips et al., 2016). In the tropical sun skink *Lampropholis coggeri,* the average hardening capacity was 0.42°C, with some individuals displaying upward shifts in heat tolerance of 2.6°C (Phillips et al., 2016). These authors also found an inverse relationship between initial CT_max_ and heat hardening, whereby skinks with higher initial heat tolerance had a lower heat hardening response than skinks with lower initial heat tolerance. This negative correlation between heat tolerance and heat hardening has been recorded for other ectotherms, including *Drosophila* (Berrigan and Hoffmann, 1998; Sørensen et al., 2001; Zatsepina et al., 2001) and porcelain crabs (Stillman, 2003). By contrast, we found no relationship between the initial CT_max_ and hardening in 6-week-old hatchlings. Nonetheless, the magnitude of the heat hardening response that we observed in velvet geckos is very similar to that reported for skinks, and suggests that like skinks, geckos have limited ability to shift their CT_max_ upwards (Phillips et al., 2016).

Interestingly, incubation under higher temperatures resulted in a significant upward shift in cold tolerance of hatchlings (Fig. 1B); aged 2 weeks, the CT_min_ of hot-incubated hatchlings was 3.3°C higher than the CT_min_ of current-incubated hatchlings. This finding mirrors the results of experimental studies on *Drosophila*. For example, in *D. melanogaster*, flies which developed at 15°C had a 4°C lower CT_min_ than flies which developed at 25°C (Slotsbo et al., 2016). Similar patterns have been reported for other species of *Drosophila* (reviewed in Hoffmann et al., 2003). While there are fewer comparable studies on lizards, a recent study on the rainforest sunskink, *Lampropholis coggeri* found that hatchlings from cool incubation (constant 23°C) had significantly lower CT_min_ at 1 month of age than hatchlings from warm (constant 26°C) incubation (Llewelyn et al., 2018). One question that arises from our study is whether the shift in cold tolerance was triggered by differences in the mean, variance or maximum temperature, since minimum temperatures in each treatment differed by only 0.7°C. In other organisms, both mean and variance in developmental temperatures can contribute to differences in cold tolerance. For example, a study on *D. melanogaster* reared flies under a warm constant environment (25°C), a warm variable environment [25±4°C (s.d.)] and a cool variable environment [18±4°C (s.d.)]. Heat tolerance of flies was unaffected by developmental temperatures, whereas chill coma recovery was longest for warm constant flies and shortest for cold variable flies (Cooper et al., 2012). However, additional studies are necessary to determine the generality of these patterns, and to elucidate the molecular pathways underpinning changes in cold tolerance.

Theoretically, developmental plasticity should result in traits that are irreversible, or at least, longer lasting than those induced via short-term heat hardening or acclimation (Piersma and Drent, 2003). To date, only one previous study on lizards has examined whether developmental plasticity for thermal tolerance persists into later life (Llewelyn et al., 2018). In a study on rainforest sunskinks, egg incubation temperature had a significant effect on the CT_min_ of hatchlings, but this difference was absent when the individuals were retested as adults (Llewelyn et al., 2018). In our study, developmental plasticity for heat tolerance was short-lived; when we retested hatchlings after 6 weeks, there was no difference in the CT_max_ of lizards from the two incubation treatments. By contrast, developmental plasticity for cold tolerance persisted into later life, and was still apparent after 6 months in the juveniles that we recaptured from our field sites. Although lizards from both incubation treatments displayed acclimation to field conditions, and shifted cold tolerance downwards, CT_min_ was still 2.32°C lower, on average, in lizards from the current-incubation treatment (Fig. 3). This pattern agrees with the results from similar studies on insects, which have found that developmental plasticity for cold tolerance is only partly reversible. For example, a study on *D. melanogaster* found that flies reared at 25°C and acclimated to 15°C as adults were able to shift their cold tolerance downwards, but still had a higher CT_min_ than 15°C reared flies after 24 days (Slotsbo et al., 2016).

The ecological consequences of developmental shifts in thermal tolerance remains poorly studied, and further research is needed to determine likely effects on survival and demography. In this study, hot-incubated eggs hatched, on average, 26 days earlier than current-incubated eggs. Thus, if nest temperatures increase in future, hatchlings will be born during mid-summer, when temperatures on rock outcrops can be lethally high during heatwaves (Dayananda et al., 2016). Whether the small developmentally-induced shifts in CT_max_ and heat hardening that we observed in the laboratory could buffer hatchlings from higher environmental temperatures requires further study. Notably, the developmental shift in CT_max_ was transient, and may therefore have little effect on survival or activity budgets. Furthermore, in most lizard species studied to date, increases in incubation temperatures tended to produce smaller hatchlings (While et al., 2018), a pattern that we also observed in this study. Therefore, developmental shifts in heat tolerance may not outweigh potential survival costs associated with a smaller body size (Andrews et al., 2000; Dayananda et al., 2017; Qualls and Andrews, 1999). Given that developmental shifts in cold tolerance were less reversible than heat tolerance, it is possible that increases in nest temperatures may produce lizards less able to cope with cold winter temperatures. For example, a study on *Anolis cristatellus* found significant downward shifts in CT_min_ between introduced and source populations, suggesting that selection has acted on this trait in natural populations (Leal and Gunderson, 2012). For our study species, winter rock temperatures routinely fall to 2.5°C in Nowra and 3°C in Dharawal (Webb, unpublished data), so lizards with lower cold tolerance may be more likely to survive cold snaps, or could have enhanced activity levels during winter. Future studies examining links between cold tolerance, heat tolerance and survival would help evaluate the demographic consequences of developmentally induced shifts in thermal tolerance.

In conclusion, we used a fluctuating temperature incubation experiment to examine the potential for developmental plasticity to produce upward shifts in the heat tolerance of hatchling velvet geckos. After maintaining hatchlings under identical conditions for 6 weeks, we found that the small increase in heat tolerance acquired from hot-temperature incubation was short-lived. Importantly, heat hardening capacity was greater in current-incubated than hot-incubated lizards, so that at 6 weeks of age, the capacity to withstand high temperatures was similar in both treatment groups. Strikingly, developmental shifts in cold tolerance were not reversible, and although both hot- and current-incubated hatchlings showed similar acclimation responses in the field, 6-month-old current-incubated lizards still had lower cold tolerance than hot-incubated lizards. Overall, our results add to the mounting body of evidence suggesting that there is little scope for developmental plasticity to buffer lizards from climate warming.

## MATERIALS AND METHODS

### Collection and maintenance of pregnant females

Gravid velvet geckos were collected from rock outcrops near Nowra, approximately 170 km south of Sydney, and Dharawal National Park, approximately 70 km south of Sydney, in late spring 2016. Females were transported to the University of Technology Sydney. Upon arrival, one of us (T.A.) measured their snout vent length (SVL) and tail length (TL) with a ruler (to the nearest millimetre), and recorded their mass (to the nearest 0.01 g) with an electronic balance. The females were housed individually inside identical plastic cages (Sistema NZ 2.0 L, 220×150×60 mm, with ventilated lid) in a constant temperature room (23°C) with a 12:12 light cycle. Each cage contained a white plastic half-pipe shelter (80 mm×40 mm) and a water dish, with a layer of moist vermiculite to prevent eggs from desiccating. Cages were placed on timer-controlled heating cables set to 32°C which created a thermal gradient (23–32°C) inside the cages during daylight hours, falling to 23°C at night. All geckos had a constant supply of drinking water and were fed crickets twice weekly. Each morning and afternoon, one of us (T.A.) checked all the cages for newly oviposited eggs, and sprayed the vermiculite to maintain a moist substrate. After females laid eggs, we recorded their body mass, and released them at their exact site of capture during suitable weather conditions.

### Egg incubation experiment

**Fig. 4.**
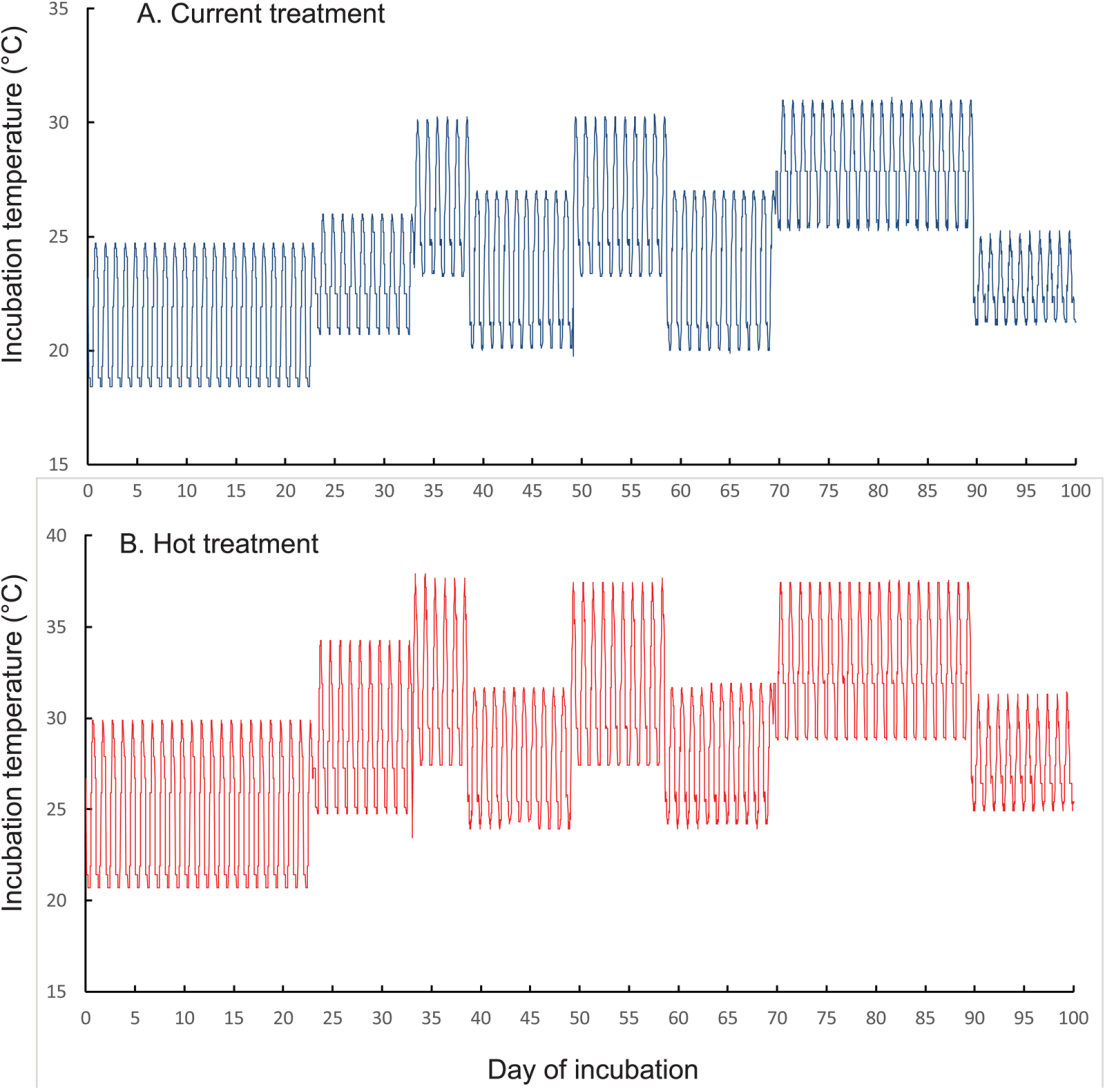
**Fluctuating temperature treatments used to incubated velvet gecko eggs.** Temperature profiles experienced by velvet gecko eggs in the (A) current- and (B) hot-incubation treatments. Both incubators were programmed to mimic the cycling temperatures that occur inside natural gecko nests, with intermittent summer heatwaves followed by cooler weather. The current treatment mimicked temperatures recorded inside sun-exposed communal nests, while the hot treatment mimicked temperatures that might occur in 2050 under climate warming.

On the day of egg laying, one of us (T.A.) placed each egg singly inside a 100 ml glass jar filled with moist vermiculite (water potential of 200 KPa), and was covered with a plastic food wrap to reduce water loss. Nearly all females laid two eggs, so we placed one egg from each clutch into the ‘current’ incubator, and the other into the ‘hot’ incubator (Panasonic MIR 154, 10 step functions). Both incubators were programmed to mimic the cycling temperatures that occur in natural nests at our study sites, but with short heatwaves to simulate a hot summer (Fig. 4). Temperature profiles of the ‘current’ treatment (mean=24.3°C, range 18.4–31.1°C, s.d.=3.2°C) were similar to those recorded inside sun-exposed communal nests (Dayananda et al., 2016), while thermal cycles of the ‘hot’ treatment (mean=28.9°C, range 19.1°C–38.1°C, s.d.=4.3°C) simulated the potential future nest temperatures that could occur in 2050 according to climate models that predict increases in air temperature between 2.9 and 4.6°C in southeast Australia (Dowdy et al., 2015). We incubated 84 eggs in the current incubation treatment and 81 eggs in the hot incubation treatment.

### Maintenance of hatchling geckos

Once hatchlings emerged from eggs, one of us (T.A.) measured their SVL with a ruler, and body mass with an electronic scale. Each hatchling was housed individually as described above for females, except that the cages were lined with a paper substrate and lacked vermiculite. We fed hatchlings with five pinhead crickets (*Gryllus assimilis*) twice weekly, and cleaned their cages at weekly intervals. All hatchlings were maintained under the same conditions in captivity for 6 weeks, after which they were released at their mothers' site of capture. All procedures were approved by the UTS Animal Care and Ethics Committee (protocol #2012000256) and a NSW National Parks and Wildlife Service scientific licence (SL101013 to J.K.W.).

### Measurements of hatchling thermal tolerance

The same researcher (T.A.) measured the thermal tolerance of 51 hatchlings (34 current incubated lizards, 17 hot incubated lizards) using the methods of Phillips et al. (2016). To measure the thermal tolerance, each lizard was placed inside an identical 50 ml plastic vial (115 mm long, 30 mm diameter) that was fitted with a removable screw cap. The vials were placed inside an incubator (Panasonic MIR 154, 10 step functions) for 30 min at 22°C to ensure that all lizards had the same starting body temperature (Terblanche et al., 2007). To commence the experiment, the vial containing a lizard was removed from the incubator, and the screw cap was replaced with a cap fitted with a thermistor thermocouple passing through its centre. The other end of the thermocouple was attached to an electronic thermometer (OMEGA 450 ATH Thermistor Thermometer 2252 Ω @25°C, accuracy 0.01°C). The thermistor was positioned so that it measured the air temperature within the tube, rather than cloacal body temperature. Because the hatchlings were very small (<0.3 g), insertion of the thermistor into the lizard's cloaca would have injured the lizards and would have prevented them from righting themselves. Given the lizards' small size, the tube temperature would provide a very close approximation of the lizard's internal temperature (Phillips et al., 2016). To commence each trial, we submerged the tube in a water bath (Thermoline) and increased the temperature gradually at a rate of 1.0°C per minute. Once the temperature reached 36°C, we checked the lizards righting response every 10 s by turning them upside down by rotating the tube. The temperature at which the lizard could not right itself was deemed the CT_max_. The same procedure was used to measure CT_min_, except that we cooled lizards from 22°C, and commenced rotating the tube to measure their righting response once they reached 18°C. All trials were carried out when hatchlings were 1–2 weeks old between 10:00 h and 15:00 h. On day 1, we measured CT_max_, and on day 2, we measured CT_min_, so that lizards had 24 h to recover between trials. Hatchlings were maintained in captivity as described above, and all lizards (except two that died in captivity) were retested at age 6 weeks.

### Measuring heat hardening capacity

**Fig. 5.**
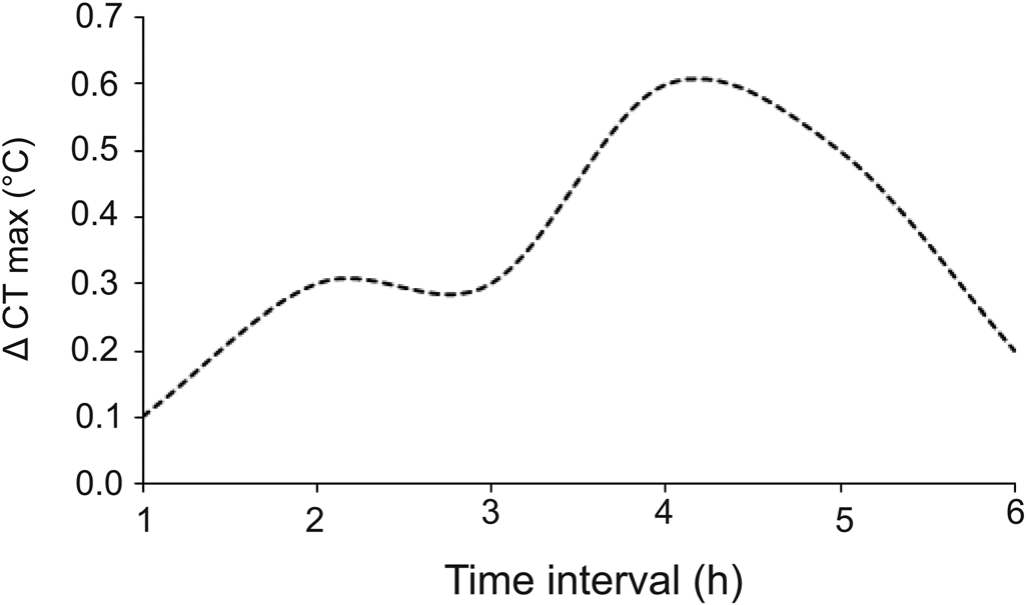
**Heat hardening (delta CT_max_) as a function of the number of hours after the first exposure to the high temperature that resulted in a loss of righting response in hatchling velvet geckos.**

We measured heat hardening of 16 current-incubated and 16 hot-incubated hatchlings at age 6 weeks. Heat hardening has not been measured in our study species previously so we first determined the time course for hardening. To do this, the same researcher (T.A.) measured the CT_max_ of a subset of lizards, as described above, and retested each individual after a period of 1–6 h. Because it can be detrimental to expose the same animal to multiple high temperatures, each individual was only tested for one time interval. The resultant curve of the change in CT_max_ (Δ CT_max_) versus time showed that maximum heat hardening occurred after 4 h (Fig. 5). Thereafter, we recorded the second measurement of CT_max_ of each lizard 4 h after the first measurement.

### Persistence of incubation induced changes in thermal tolerance

To determine whether thermal tolerance changed with age under laboratory conditions, the same researcher (T.A.) retested 49 lizards (33 current-incubated, 16 hot-incubated) at 6 weeks of age. All lizards were raised under the same environmental conditions (see above) in the laboratory prior to testing. After testing was completed, we individually marked each lizard with a unique toe-clip, and released them at their mothers’ site of capture. In mid-winter (July), we systematically searched under all the rocks at our field sites and checked the toe-clips of all geckos captured. The hatchlings from our incubation experiment that we recaptured were brought to the laboratory, and housed as described previously. We measured the thermal tolerance and heat hardening of these lizards within 1 day of capture using the methods described above. After testing was complete, the lizards were returned to their exact site of capture.

### Statistical analyses

A chi-square test was used to determine whether hatchling success varied between incubation treatments. Two-factor ANOVAs were used to determine whether incubation period or body size SVL differed between sites or treatments. We used linear mixed effects models to determine whether CT_max_, CT_min_ and heat hardening differed between hot-incubated and current-incubated lizards. Each model had either CT_max_, CT_min_ or heat hardening as the response variable. Incubation temperature was a fixed explanatory variable (current, hot) and both location (Dharawal National Park, Nowra) and mother identity were included as random control variables. Mother identity was nested within location in the models. Heat hardening was log_e_-transformed prior to analyses to meet assumptions of normality and homogeneity of variance in the model residuals. Satterthwaite approximations were used to calculate degrees of freedom for *t*-tests in the mixed models and *P*-values were calculated using the adjusted degrees of freedom. Statistical analyses were performed using the package lmerTest (Kuznetsova et al., 2017) in R 3.1.3 (https://www.r-project.org/).
